# Constructing a predictive model for the risk of periventricular leukomalacia in very low birth weight premature infants based on Lasso-Logistic regression analysis

**DOI:** 10.3389/fped.2026.1651586

**Published:** 2026-09-03

**Authors:** Faxiu Duan, LiZhen Ling, Lijuan Liao

**Affiliations:** Department of Neonatology, Ganzhou People’s Hospital, Ganzhou, Jiangxi, China

**Keywords:** lasso-Logistic regression, low birth weight premature infants, nomogram model, periventricular leukomalacia, predictors

## Abstract

**Objective:**

To analyze the predictors of periventricular leukomalacia (PVL) in very low birth weight premature infants based on Lasso-Logistic regression and construct a nomogram prediction model.

**Methods:**

From March 2022 to May 2024, 248 very low birth weight premature infants born and hospitalized in our hospital were marked as the modeling group. Another 106 very low birth weight premature infants hospitalized from June 2024 to March 2025 were selected as the verification group. Lasso Logistic regression was used to screen for the predictors of PVL in very low birth weight premature infants. The nomogram risk prediction model was constructed. Hosmer-Lemeshow test and ROC analysis were used to evaluate the predictive value of the model. The decision curve analysis (DCA) was used to evaluate the clinical application value of the model.

**Results:**

The total incidence of PVL in 354 premature infants was 23.73%. Lasso and logistic regression results showed that Apgar score at 1 min after birth (OR = 0.645), C-reactive protein (CRP) (OR = 5.687), neonatal respiratory distress syndrome (NRDS) (OR = 5.412), sepsis (OR = 6.752), patent ductus arteriosus (OR = 9.428), and chorioamnionitis (OR = 8.342) were the predictors of PVL in very low birth weight premature infants (*P* < 0.05). ROC analysis and Hosmer-Lemeshow test suggested that the model had high predictive consistency and discrimination, while the DCA curve suggested that this model had high clinical application value.

**Conclusion:**

The risk of PVL in very low birth weight premature infants is closely related to Apgar score at 1 min after birth, CRP, NRDS, sepsis, patent ductus arteriosus, and chorioamnionitis. The model established based on these six factors has good predictive performance.

## 1 Introduction

Periventricular leukomalacia (PVL) is a type of brain injury, primarily caused by hypoxia-ischemia, and commonly occurs in preterm infants (1). The pathogenesis of PVL involves multiple aspects, including hypoxia-ischemia, inflammatory response, and vascular structural abnormalities. Its main clinical manifestations include abnormal muscle tone, seizures, and ocular abnormalities. Some affected infants, although showing no obvious symptoms in the early stages, may later develop cognitive and intellectual abnormalities (2, 3). Preterm infants with mild PVL have a possibility of cure with timely intervention in the early stage of onset, but severe cases are difficult to fully recover. If not diagnosed in time, it will affect treatment efficacy and lead to a poor prognosis (4). Exploring the predictors of PVL in preterm infants is conducive to timely clinical diagnosis and implementation of intervention measures, thereby improving the quality of life of the affected infants. Currently, some studies have analyzed the predictors of PVL in preterm infants, but simply analyzing these factors cannot predict the risk of PVL occurrence (5). A nomogram is a model constructed based on multivariate Logistic regression. This model can intuitively display the magnitude of the impact of multiple predictors on outcome events using scores and is widely used in clinical research (6, 7). Since the incidence of PVL is higher in very low birth weight (VLBW) preterm infants (8), this study analyzes the predictors of PVL in VLBW preterm infants and constructs a nomogram model to facilitate timely clinical diagnosis of PVL.

## 2 Materials and methods

### 2.1 General data

**Figure 1:**
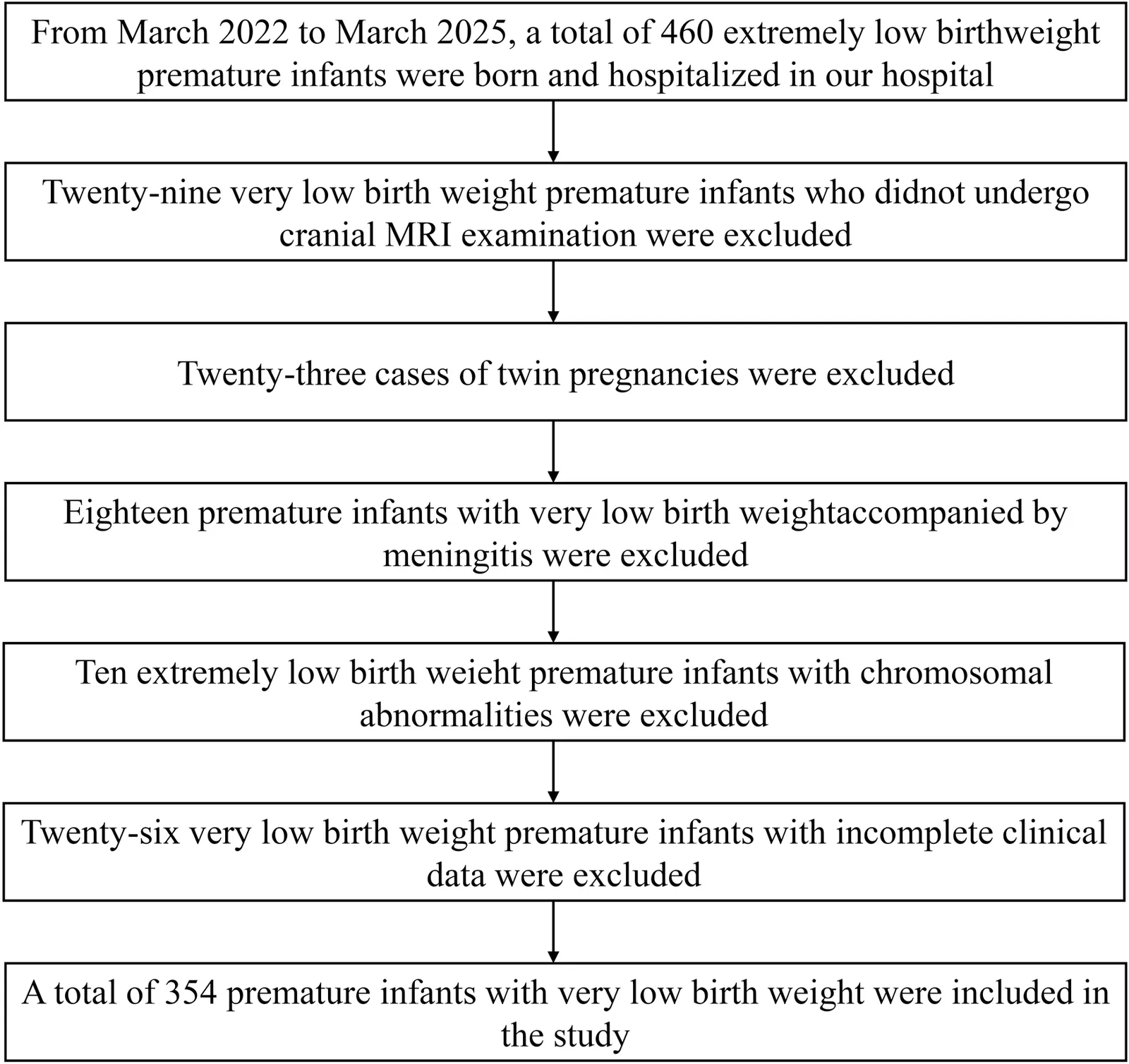
Flowchart of case collection.

This retrospective study was conducted based on the sample size calculation formula *N* = Z^2^ × [*P* × (1−P)]/E^2^, with Z = 1.96, E = 0.044, and *P* = 0.15, the required sample size was *N* = 248. Therefore, A total of 248 very low birth weight (VLBW) preterm infants born and hospitalized in our hospital from March 2022 to May 2024 were selected as the modeling group, and another 106 VLBW preterm infants born and hospitalized from June 2024 to March 2025 were selected as the validation group. Inclusion criteria: (1) 28 weeks ≤ gestational age <37 weeks; (2) birth weight <1,500 g, classified as very-low-birth-weight preterm infants; (3) underwent cranial MRI examination during hospitalization, and the brain MRI findings were interpreted by qualified specialist physicians. Exclusion criteria: (1) twin pregnancy; (2) meningitis; (3) chromosomal abnormalities; (4) incomplete clinical data. Figure 1 shows the case collection flowchart. This study was approved by the Medical Ethics Committee.

### 2.2 Methods

#### 2.2.1 PVL assessment

All preterm infants underwent MRI examination within two weeks of birth. The diagnostic criteria for PVL referenced literature were based on reference (9), and the MRI findings were interpreted by radiologists blinded to the clinical status.

#### 2.2.2 Data collection

Collected clinical data included sex of the preterm infant, birth weight, 1-minute Apgar score, routine blood indicators detected at birth (umbilical cord blood) such as white blood cell count and C-reactive protein (CRP), intrauterine infection (yes/no), intrauterine distress (yes/no), neonatal respiratory distress syndrome (NRDS), patent ductus arteriosus (yes/no), neonatal pneumonia (yes/no), as well as mode of delivery (vaginal delivery/cesarean section), and maternal history of gestational diabetes mellitus and hypertension.

### 2.3 Statistical analysis

SPSS 27.0 and R 4.3.1 were used for data analysis and figure generation. Count data were expressed as *n* (%) and analyzed using the chi-square test. Apgar scores, being measurement data not conforming to normal distribution, were expressed as M (P25, P75) and analyzed using the Mann–Whitney U test. Other normally distributed measurement data were expressed as mean ± standard deviation (mean ± SD) and analyzed using the t-test. After performing Lasso analysis in R software, multivariate Logistic regression analysis was conducted in SPSS to screen for predictors of PVL in VLBW preterm infants, followed by the Hosmer-Lemeshow test and receiver operating characteristic (ROC) analysis; a nomogram risk prediction model was constructed and a calibration curve was plotted using online nomogram creation software; Clinical Decision Curve Analysis (DCA) was plotted in R software. A *P*-value <0.05 was considered statistically significant.

## 3 Results

### 3.1 Incidence of PVL in preterm infants

In the modeling group of 248 preterm infants, 62 developed PVL. In the validation group of 106 preterm infants, 22 developed PVL. There was no significant difference in the incidence of PVL between the two groups (25.00% vs. 20.75%) (*χ*^2^ = 0.739, *P* = 0.390). The overall incidence of PVL in 354 preterm infants was 23.73%.

### 3.2 Comparison of maternal and preterm infant data between the modeling and validation groups

There were no significant differences in the preterm infants' own data or maternal data between the two groups (*P* > 0.05). See Table 1.

**Table 1:** Compares the maternal and fetal data of the modeling group and the verification group [*n* (%)/ ± ].

Indexs	*n*	Modeling Group (*n* = 248)	Verification group (*n* = 106)	*t*/*Z*/*χ*^2^	*P*
Premature infants' own factors					
Gender				0.265	0.607
Male	193	133 (53.63)	60 (56.60)		
Female	161	115 (46.37)	46 (43.40)		
Gestational age (weeks)	–	31.94 ± 1.62	31.71 ± 1.54	1.241	0.215
Birth weight (g)	–	1,379.60 ± 140.67	1,395.36 ± 135.83	0.975	0.330
Apgar score at 1 min after birth (points)	–	7 (6, 8)	7 (6, 8)	0.726	0.389
White blood cell count (×10^9^/L)	–	12.62 ± 2.09	12.84 ± 2.17	0.897	0.370
Platelet count (×10^9^/L)	–	230.61 ± 38.69	225.19 ± 36.42	1.228	0.220
Red blood cell count (×10^12^/L)	–	6.22 ± 1.24	6.41 ± 1.28	1.308	0.192
CRP (mg/L)	–	1.36 ± 0.35	1.30 ± 0.37	1.452	0.147
Neonatal anemia				0.055	0.814
Yes	32	23 (9.27)	9 (8.49)		
No	322	225 (90.73)	97 (91.51)		
Intrauterine infection				0.971	0.325
Yes	26	16 (6.45)	10 (9.43)		
No	328	232 (93.55)	96 (90.57)		
Intrauterine distress				0.930	0.335
Yes	35	27 (10.89)	8 (7.55)		
No	319	221 (89.11)	98 (92.45)		
NRDS				0.453	0.501
Yes	82	55 (22.18)	27 (25.47)		
No	272	193 (77.82)	79 (74.53)		
Sepsis				0.071	0.789
Yes	46	33 (13.31)	13 (12.26)		
No	308	215 (86.69)	93 (87.74)		
Patent ductus arteriosus				0.122	0.727
Yes	21	14 (5.65)	7 (6.60)		
No	333	234 (94.35)	99 (93.40)		
Neonatal necrotizing enterocolitis				0.104	0.747
Yes	18	12 (4.84)	6 (5.66)		
No	336	236 (95.16)	100 (94.34)		
Neonatal pneumonia				0.457	0.499
Yes	50	33 (13.31)	17 (16.04)		
No	304	215 (86.69)	89 (83.96)		
Maternal factor					
Mode of delivery				0.517	0.472
Natural childbirth	120	87 (35.08)	33 (31.13)		
Cesarean section	234	161 (64.92)	73 (68.87)		
Hypertension in pregnancy				0.299	0.585
Yes	77	52 (20.97)	25 (23.58)		
No	277	196 (79.03)	81 (76.42)		
Gestational diabetes mellitus				0.485	0.486
Yes	61	45 (18.15)	16 (15.09)		
No	293	203 (81.85)	90 (84.91)		
Placental insufficiency				0.139	0.709
Yes	102	70 (28.23)	32 (30.19)		
No	252	178 (71.77)	74 (69.81)		
Chorioamnionitis				0.034	0.855
Yes	45	31 (12.50)	14 (13.21)		
No	309	217 (87.50)	92 (86.79)		

### 3.3 Univariate analysis of periventricular leukomalacia in the modeling group

Compared to the non-PVL group, the PVL group had lower 1 min Apgar scores, higher CRP levels, and higher proportions of NRDS, sepsis, patent ductus arteriosus, and chorioamnionitis (*P* < 0.05). See Table 2.

**Table 2:** Comparison of data of premature infants and their mothers in the non-occurrence group and the occurrence group [*n* (%)/ ± ].

Indexs	*n*	Non-occurrence group (*n* = 186)	Occurrence group (*n* = 62)	*t*/*Z*/*χ*^2^	*P*
Premature infants' own factors					
Gender				0.265	0.607
Male	133	98 (52.69)	35 (56.45)		
Female	115	88 (47.31)	27 (43.55)		
Gestational age (weeks)	–	32.05 ± 1.62	31.63 ± 1.57	1.781	0.076
Birth weight (g)	–	1,386.49 ± 138.15	1,358.91 ± 141.32	1.354	0.177
Apgar score at 1 min after birth (points)	–	8 (7, 9)	7 (6, 8)	4.078	<0.001
White blood cell count (×10^9^/L)	–	12.71 ± 2.11	12.35 ± 2.06	1.170	0.243
Platelet count (×10^9^/L)	–	231.54 ± 37.50	227.82 ± 35.49	0.685	0.494
Red blood cell count (×10^12^/L)	–	6.25 ± 1.26	6.14 ± 1.21	0.601	0.548
CRP (mg/L)	–	1.31 ± 0.28	1.54 ± 0.38	5.095	<0.001
Neonatal anemia				1.294	0.255
Yes	23	15 (8.06)	8 (12.90)		
No	225	171 (91.94)	54 (87.10)		
Intrauterine infection				0.802	0.371
Yes	16	10 (5.38)	6 (9.68)		
No	232	176 (94.62)	56 (90.32)		
Intrauterine distress				2.341	0.126
Yes	27	17 (9.14)	10 (16.13)		
No	221	169 (90.86)	52 (83.87)		
NRDS				10.661	0.001
Yes	55	32 (17.20)	23 (37.10)		
No	193	154 (82.80)	39 (62.90)		
Sepsis				8.494	0.004
Yes	33	18 (9.68)	15 (24.19)		
No	215	168 (90.32)	47 (75.81)		
Patent ductus arteriosus				6.460	0.011
Yes	14	6 (3.23)	8 (12.90)		
No	234	180 (96.77)	54 (87.10)		
Neonatal necrotizing enterocolitis				1.051	0.305
Yes	12	7 (3.76)	5 (8.06)		
No	236	179 (96.24)	57 (91.94)		
Neonatal pneumonia				2.622	0.105
Yes	33	21 (11.29)	12 (19.35)		
No	215	165 (88.71)	50 (80.65)		
Maternal factor					
Mode of delivery				1.706	0.192
Natural childbirth	87	61 (32.80)	26 (41.94)		
Cesarean section	161	125 (67.20)	36 (58.06)		
Hypertension in pregnancy				3.244	0.072
Yes	52	34 (18.28)	18 (29.03)		
No	196	152 (81.72)	44 (70.97)		
Gestational diabetes mellitus				2.036	0.154
Yes	45	30 (16.13)	15 (24.19)		
No	203	156 (83.87)	47 (75.81)		
Placental insufficiency				3.211	0.073
Yes	70	47 (25.27)	23 (37.10)		
No	178	139 (74.73)	39 (62.90)		
Chorioamnionitis				13.382	<0.001
Yes	31	15 (8.06)	16 (25.81)		
No	217	171 (91.94)	46 (74.19)		

### 3.4 Lasso and multivariate logistic regression analysis of PVL in very Low birth weight preterm infants

**Figure 2:**
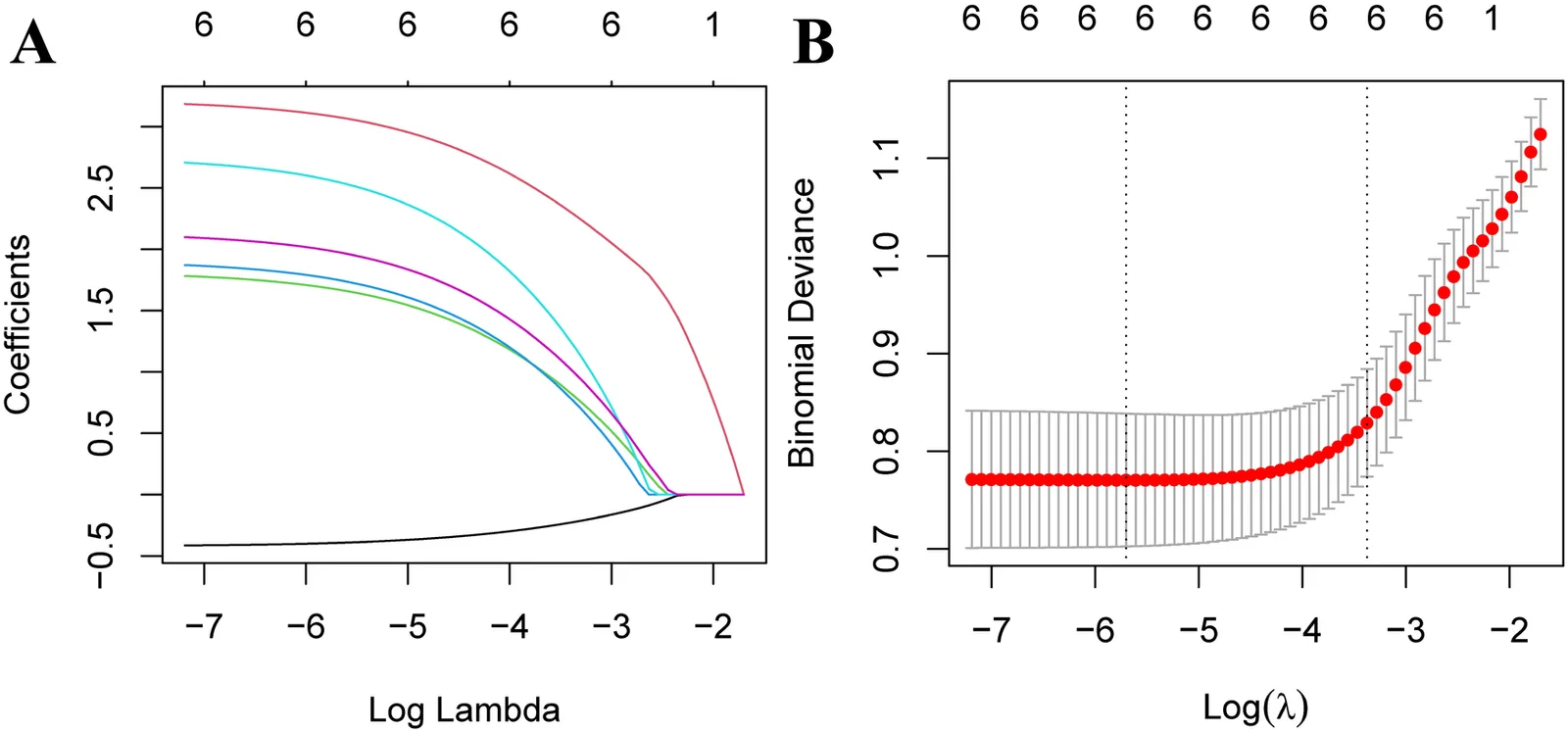
Flowchart of the *λ* selection and variable selection process **(A)** diagram of the cross-validation optimal parameter *λ* selection process; **(B)** dynamic process diagram of lasso regression screening variables.

Lasso regression analysis was performed with the occurrence of PVL in the modeling group (occurrence = 1, non-occurrence = 0) as the dependent variable, and 1 min Apgar score (continuous variable), CRP level (continuous variable), NRDS (yes = 1, no = 0), sepsis (yes = 1, no = 0), patent ductus arteriosus (yes = 1, no = 0), and chorioamnionitis (yes = 1, no = 0) as independent variables. The results showed that all 6 predictors were selected, and the model performance was optimal when the penalty coefficient *λ* = 0.0342075. The *λ* selection process and variable screening process diagrams are shown in Figure 2.

**Figure 3:**
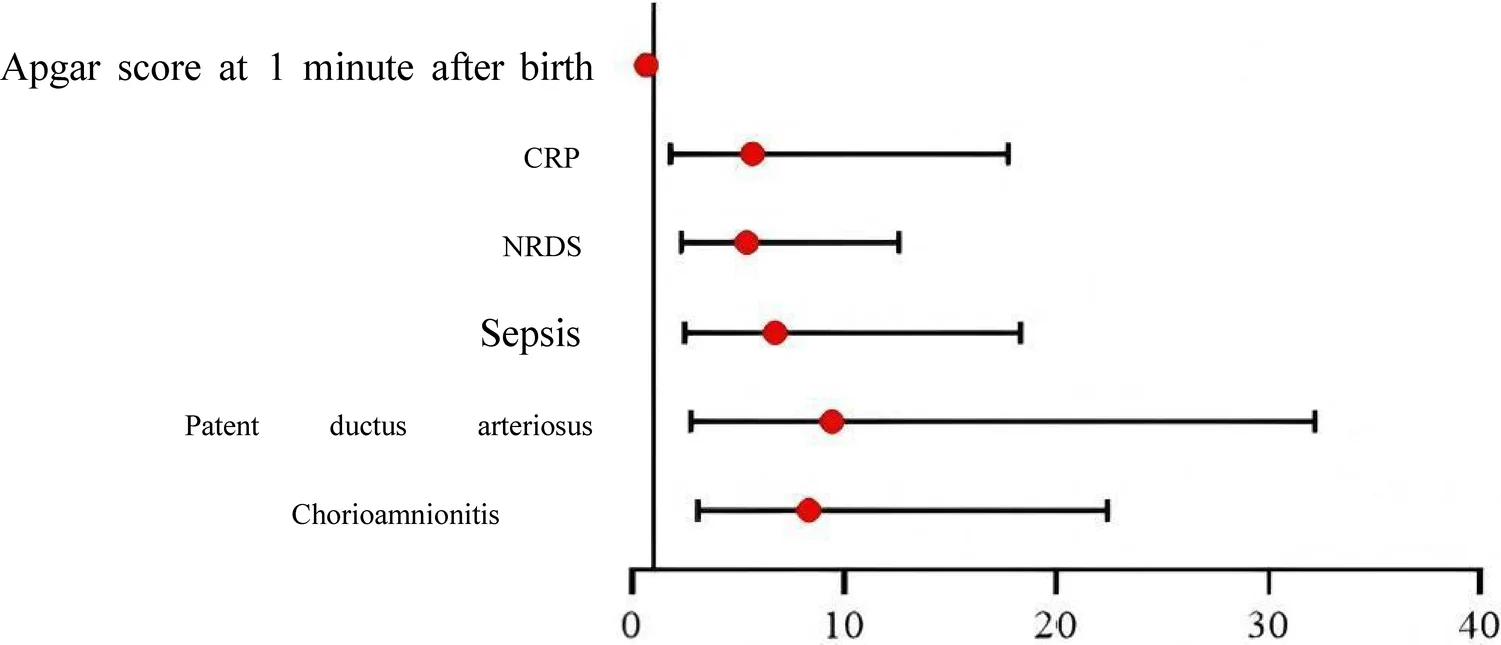
Forest map.

These 6 factors were further included as independent variables in a multivariate Logistic regression. The results showed that a high 1 min Apgar score at birth (OR = 0.645), elevated CRP (OR = 5.687), NRDS (OR = 5.412), sepsis (OR = 6.752), patent ductus arteriosus (OR = 9.428), and chorioamnionitis (OR = 8.342) were factors influencing PVL development PVL (*P* < 0.05). See Table 3. Figure 3 shows a forest plot based on the Logistic regression results.

**Table 3:** Results of multivariate logistic regression analysis.

Variables	*β*	*SE*	Wald *χ*^2^	*P*	*OR*	95% *CI*
Apgar score at 1 min after birth	−0.439	0.128	11.682	0.001	0.645	0.501–0.829
CRP	1.738	0.581	8.941	0.003	5.687	1.820–17.772
NRDS	1.689	0.432	15.275	<0.001	5.412	2.320–12.620
Sepsis	1.91	0.51	14.026	<0.001	6.752	2.485–18.343
Patent ductus arteriosus	2.244	0.627	12.822	<0.001	9.428	2.761–32.193
Chorioamnionitis	2.121	0.503	17.752	<0.001	8.342	3.110–22.378
Constant term	−1.778	1.239	2.059	0.151	0.169	–

### 3.5 Nomogram model for predicting the risk of PVL in very low birth weight preterm infants

**Figure 4:**
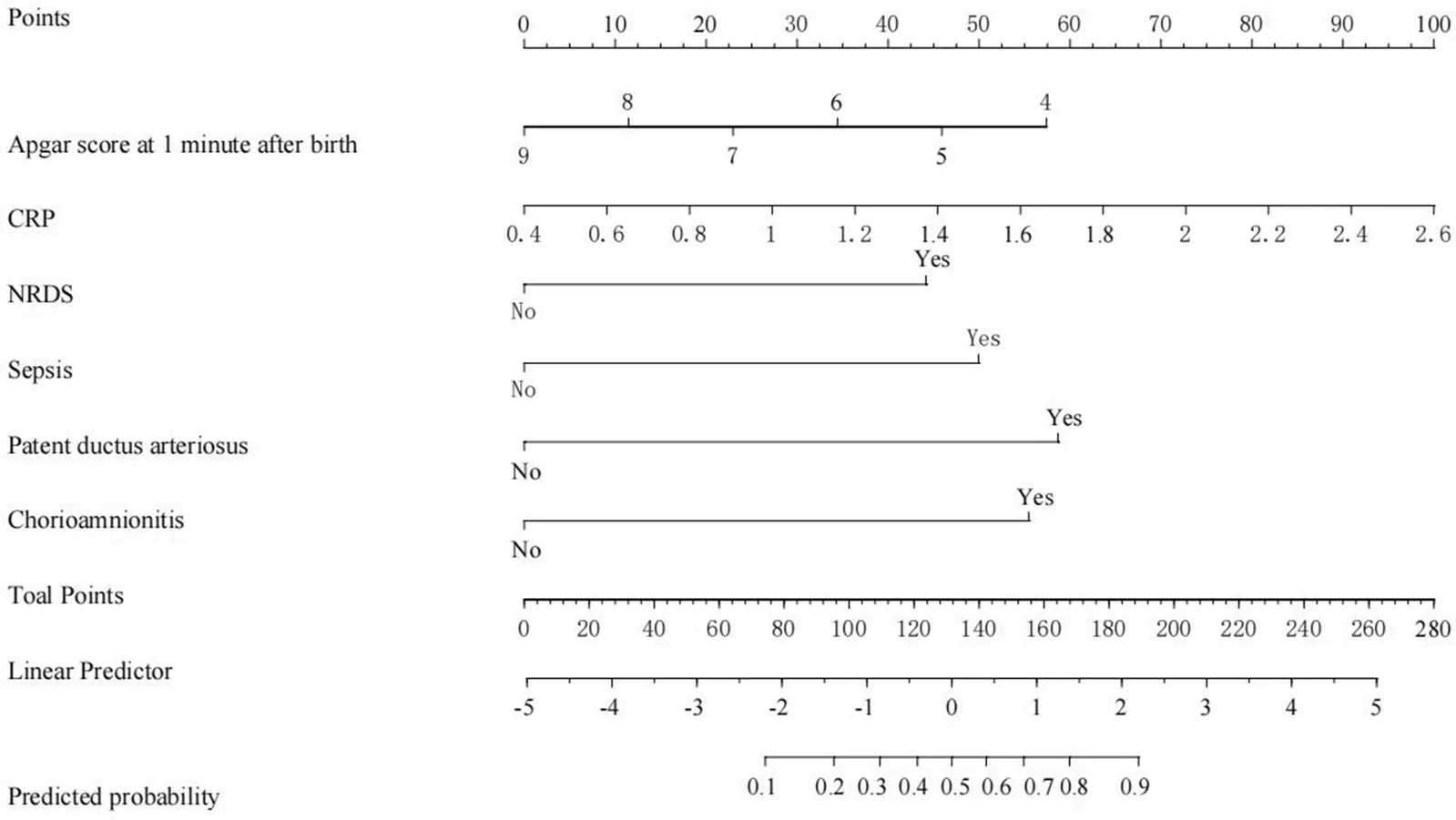
Nomogram model.

A nomogram model for predicting the risk of PVL was established based on the above 6 factors. The results showed that lower 1-minute Apgar scores and higher CRP levels resulted in higher scores; NRDS added a weight of 44 points, sepsis added a weight of 50 points, patent ductus arteriosus added a weight of 59 points, and chorioamnionitis added a weight of 55 points. Total scores of 154, 168, and 189 corresponded to predicted probability values of 0.7, 0.8, and 0.9, respectively. See Figure 4.

### 3.6 Evaluation of model predictive performance

**Figure 5:**
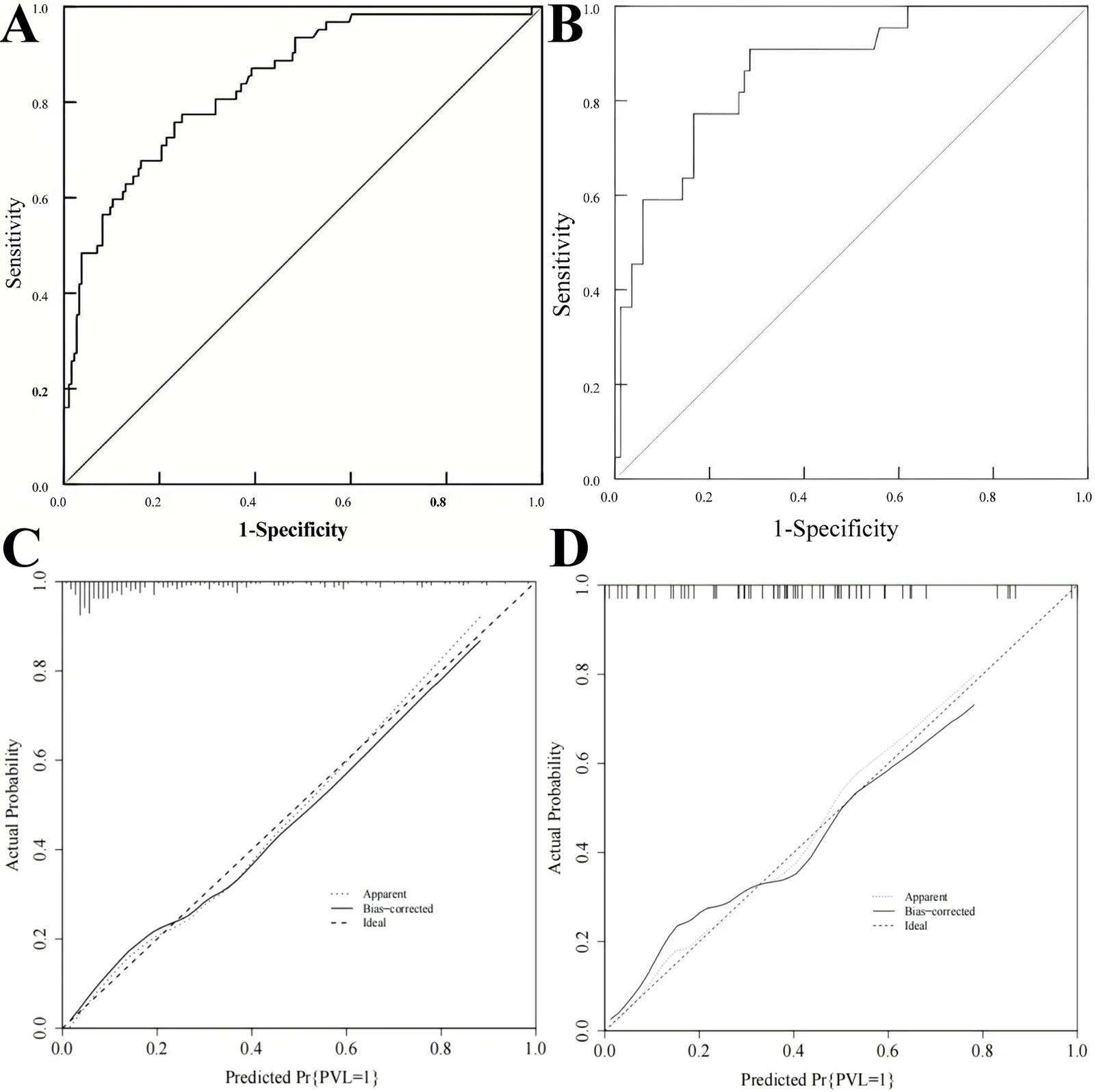
Evaluation of model predictive performance **(A)** ROC curve of modeling group; **(B)** ROC curve of validation group; **(C)** calibration curve of modeling group; **(D)** calibration curve of validation group.

ROC analysis showed that the AUC for the modeling group was 0.841 (95% CI: 0.785–0.898), and the AUC for the validation group was 0.865 (95% CI: 0.783–0.947). The Hosmer-Lemeshow test showed *χ*^2^ = 7.060, *P* = 0.530 for the modeling group, and *χ*^2^ = 5.072, *P* = 0.750 for the validation group. Furthermore, the calibration curve showed good agreement between the actual curve and the ideal curve. See Figure 5.

### 3.7 Evaluation of the clinical application value of the model

**Figure 6:**
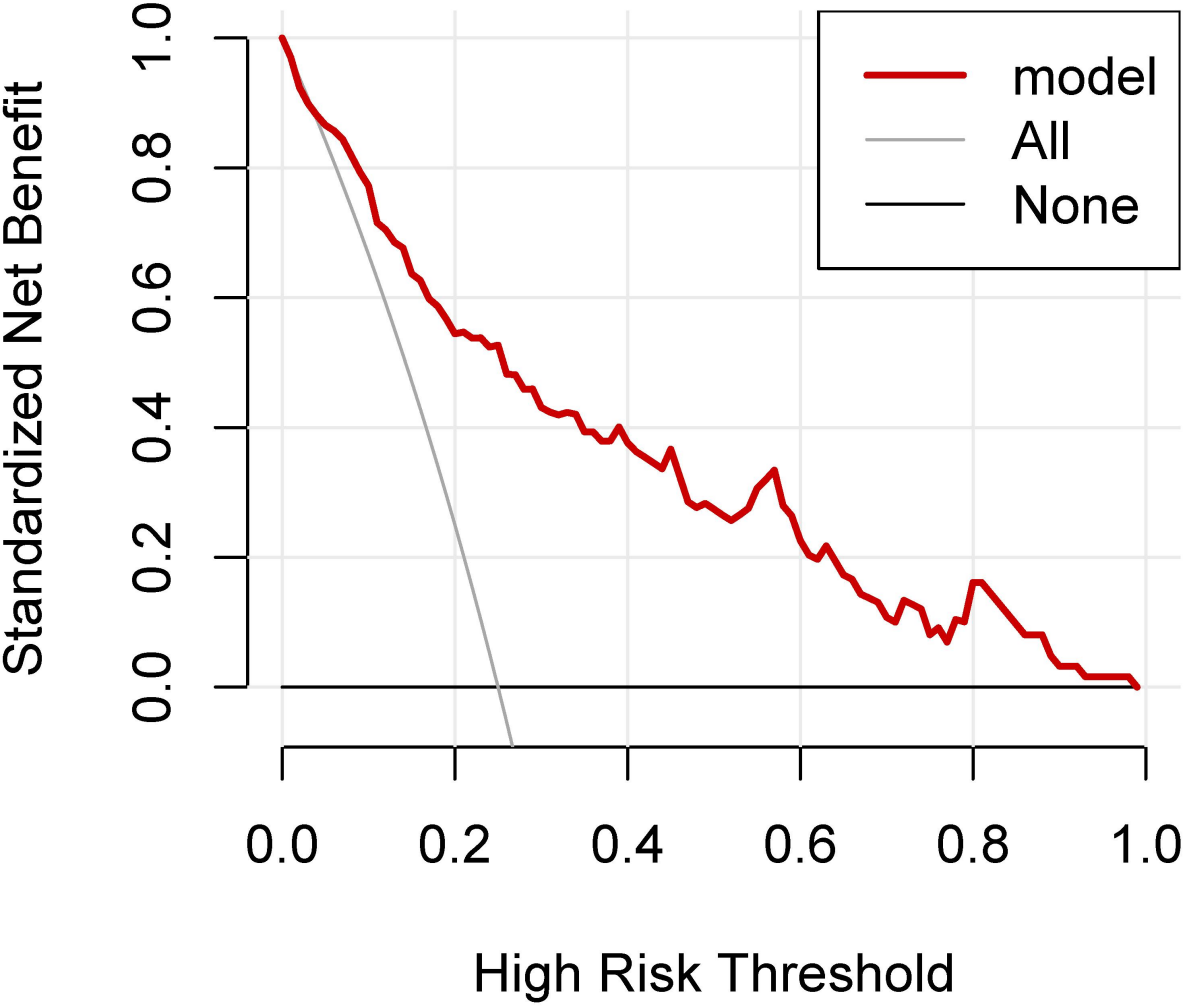
DCA curve.

The DCA curve showed that when the high-risk threshold of the model was in the range of 0.02–1.0, the clinical net benefit was relatively high and could be used to guide clinical decision-making. See Figure 6.

## 4 Discussion

PVL poses multifaceted harm to preterm infants. Brain injury and retinopathy are equally important risk factors for blindness in preterm infants (10). A study by Yazici et al. (11) showed that PVL is associated with neurodevelopmental impairment in very low birth weight preterm infants with moderate/severe bronchopulmonary dysplasia. Other research (12) has also reported that PVL is one of the risk factors for language delay in very low birth weight preterm infants. Currently, treatment for PVL includes the use of neurotrophic factors and other drugs, as well as providing nutritional support. Timely diagnosis is of positive significance for improving the prognosis of preterm infants with PVL (13). Establishing a predictive model can help screen high-risk preterm infants for PVL in advance, enabling timely diagnosis.

Among the 354 very low birth weight preterm infants included in this study, 84 developed PVL, indicating a relatively high incidence (23.73%). The overall incidence of PVL in the present cohort was 23.73%, which was relatively high compared with some previous reports. This discrepancy may be explained by differences in study population, diagnostic methods, and disease definition. The present study included hospitalized very-low-birth-weight preterm infants who underwent cranial MRI within two weeks after birth. MRI may be more sensitive than cranial ultrasonography for detecting early-stage or non-cystic white matter injury. In addition (14), the PVL group had higher proportions of NRDS, sepsis, patent ductus arteriosus, and chorioamnionitis, which may also contribute to the increased incidence of PVL in this cohort (15). Therefore, the incidence observed in this study should be interpreted in the context of the high-risk clinical characteristics of the included population. The univariate analysis conducted in this study showed that there were differences in 1-minute Apgar scores and CRP levels between VLBW preterm infants who developed PVL and those who did not. Furthermore, among preterm infants who developed PVL, the proportions of those with NRDS, sepsis, patent ductus arteriosus, and chorioamnionitis were higher. Through subsequent Lasso and Logistic regression analyses, we concluded that 1-minute Apgar score, CRP level, NRDS, sepsis, patent ductus arteriosus, and chorioamnionitis are predictors for PVL in VLBW preterm infants. Previous research (15) has also indicated that sepsis, patent ductus arteriosus, and chorioamnionitis are closely related to the occurrence of PVL in preterm infants. Analyzing the reasons, a lower Apgar score at birth indicates more severe perinatal hypoxia-ischemia. Due to the immature development of cerebral blood vessels in preterm infants, with fewer vascular branches around the ventricles, they are susceptible to the effects of hypoxia-ischemia, thereby causing PVL (16). Higher CRP levels in preterm infants suggest possible bacterial infection, and inflammatory factors can lead to oligodendrocyte apoptosis and interfere with myelination, thus leading to the occurrence of PVL (17). NRDS is caused by a lack of pulmonary surfactant, and its occurrence can cause systemic hypoxia, increasing the risk of PVL. Sepsis is accompanied by a strong inflammatory response and oxidative stress, which can damage the blood-brain barrier and is therefore closely related to PVL (18). Patent ductus arteriosus will lead to blood shunting from the aorta to the pulmonary artery. This hemodynamic change can cause insufficient cerebral perfusion, leading to damage to the periventricular white matter (19). The occurrence of chorioamnionitis can activate the maternal and fetal inflammatory responses. Inflammatory factors cross the placental barrier and affect the fetal brain tissue, damaging white matter cells (20).

This study constructed a nomogram model based on relevant predictors. The model shows that the higher the total score, the higher the risk of PVL occurrence. Clinically, the risk of PVL in VLBW preterm infants can be predicted based on the scores of the above six factors, and timely diagnosis can be made for preterm infants with high-risk values. This study further used various methods to evaluate the predictive performance of the model. The ROC curve indicated that the model had a high degree of discrimination between PVL and non-PVL in both the internal and validation datasets. The Hosmer-Lemeshow test and calibration curve indicated that the predicted probability values of the model were close to the actual probability values, i.e., good consistency. In addition, in the DCA curve, the clinical net benefit was high over a long range of high-risk thresholds, indicating good clinical application value. The nomogram model constructed in this study is not only of great significance for the timely diagnosis of PVL but also has positive significance for reducing the risk of PVL in VLBW preterm infants. Based on the analysis results of Razak et al. (21), it is speculated that the risk of PVL in preterm infants can be reduced through interventions such as the use of erythropoiesis-stimulating agents, nutritional support, and delayed cord clamping. Early identification of PVL may enable a multidisciplinary team to develop individualized inpatient rehabilitation and post-discharge follow-up plans, thereby optimizing long-term neurodevelopmental outcomes.

In summary, the nomogram model constructed based on the factors of 1-minute Apgar score, CRP, NRDS, sepsis, patent ductus arteriosus, and chorioamnionitis for predicting the risk of PVL in very low birth weight preterm infants has relatively good predictive performance and certain clinical application value. However, this study still has certain limitations, including a limited sample size, its single-center and retrospective design, and the lack of investigation of long-term neurodevelopmental outcomes; therefore, the generalization performance of the model still needs further verification.

## Data Availability

The original contributions presented in the study are included in the article/Supplementary Material, further inquiries can be directed to the corresponding author.
